# Study of Canine Distemper Virus Presence in Catalonia’s Wild Carnivores through H Gene Amplification and Sequencing

**DOI:** 10.3390/ani14030436

**Published:** 2024-01-29

**Authors:** Junhao Huang, Martí Cortey, Laila Darwich, Jenna Griffin, Elena Obón, Rafael Molina, Margarita Martín

**Affiliations:** 1 Department of Animal Health and Anatomy, Autonomous University of Barcelona (UAB), 08193 Cerdanyola del Vallès, Spain; junhao.huang@autonoma.cat (J.H.); marti.cortey@uab.cat (M.C.); laila.darwich@uab.cat (L.D.); jennabgriffin6@gmail.com (J.G.); 2 Torreferrussa Wildlife Rehabilitation Centre, Catalan Wildlife Service-Forestal Catalana S.A., 08130 Santa Perpètua de Mogoda, Spain; elena.obon@gencat.cat

**Keywords:** red fox (*Vulpes vulpes*), badger (*Meles meles*), American mink (*Neogale vison*), Eurasian otter (*Lutra lutra*), canine distemper virus (CDV)

## Abstract

**Simple Summary:**

Several red foxes in Spain have recently been identified as testing positive for canine distemper virus (CDV). This study focused on the presence of CDV in wild carnivores in Catalonia (north-eastern Spain) for conservation aims, including the red fox (*Vulpes vulpes*), badger (*Meles meles*), American mink (*Neogale vison*) and Eurasian otter (*Lutra lutra*). The results confirmed the circulation of CDV in Catalonia, revealing a notable percentage of positive cases in red foxes and European badgers. Phylogenetic and spatial studies emphasise the risk of CDV transmission among carnivores in this region likely due to potential reservoirs, close interactions, and shared environmental spaces. Furthermore, the circulation of CDV in wildlife represents a threat to endangered species such as the European polecat (*Mustela putorius*).

**Abstract:**

Canine distemper virus (CDV) is recognised worldwide as an important pathogen in both domestic and wild carnivores. Few data are available on its impact and spread on the wildlife/wildlife–domestic animal–environment interface. This study, aimed at developing a conservation-oriented control strategy, analysed 89 sick or deceased animals from 2019 to 2023 at the Wildlife Rehabilitation Centre in Torreferrussa. RT-PCR and sequencing of the partial H gene were used to detect and analyse CDV in tissues. The total positive percentage was 20.22% (18/89), comprising 13 red foxes (44.8%), 4 European badgers (28.6%), and 1 American mink (4.5%), while 24 Eurasian otters tested negative. Phylogenetic analysis indicated that all of the CDV strains belong to the European lineage. Geographically distant individuals and different species shared the same viral strain, suggesting a strong capacity of CDV for interspecies and long-distance transmission. This calls for further research, particularly focusing on potential impacts of CDV on endangered carnivores.

## 1. Introduction

Canine distemper virus (CDV), along with the measles and rinderpest viruses, is a member of the *Morbillivirus* genus in the *Paramyxoviridae* family. It is an enveloped virus that has a single negative-stranded RNA genome. The CDV genome includes six structural proteins: matrix (M), fusion (F), haemagglutinin (H), nucleocapsid (N), polymerase (L), and phosphoprotein (P) [1]. The H protein of *Morbillivirus* helps the virus adsorb to the receptor on the cell surface. Therefore, the H protein determines the specificity of the CDV host and assists the F protein to make CDV enter the host cell by fusion of the envelope and the host cell membrane. In particular, due to frequent mutations of the H gene, it is a valuable marker for studying genetic variations among different strains of CDV [2]. Generally, CDV is considered to have only one single serotype, but by sequencing the H gene, it was determined to have at least 18 major genetic lineages: Asia-1 to Asia-5, America-1, America-2, North America-3, South America/North America-4, America-5, Africa-1, Africa-2, Europe-1/South America-1, South America-2, South America-3, Europe Wildlife, Arctic, and Rockborn-like [3,4,5,6].

The virus’s route of transmission is entrance into the body through oral or nasal routes. In the tonsils and the upper respiratory tract, CDV multiplies in macrophages, demonstrating a strong affinity for lymphoid tissues [7]. Additionally, by infecting dendritic cells that transport the virus to nearby lymph nodes, CDV infects activated T and B cells. It then spreads through the lymphatic system to secondary lymphoid organs and targets tissues including the central nervous system (CNS), respiratory tract, gastrointestinal tract, uvea, urothelium, skin, kidney, and liver [7,8].

Clinical presentations associated with CDV include biphasic fever, a progression of serous ocular–nasal discharge to mucopurulent discharge, anorexia, conjunctivitis, bronchitis, pneumonia, gastroenteritis, neurological signs, and skin lesions such as vesicular or pustular dermatitis or hyperkeratosis [9,10,11]. Histologically, CDV induced lymphoid tissue necrosis in respiratory, urogenital, gastrointestinal epithelial cells, Kupffer cells, gametocytes, glial cells, and neurons, leading to interstitial pneumonia and the formation of intracytoplasmic and intranuclear inclusions [12,13,14]. To confirm the presence of CDV, various laboratory techniques can be utilised, including an enzyme-linked immunosorbent assay (ELISA) [15], haematology [16], histopathology [13], immunocytology [17], immunohistochemistry [18], nucleic acid detection [19], virus isolation [20], and virus neutralisation tests [21].

Although vaccination in domestic dogs is effective, immunising non-domestic carnivores against CDV has introduced challenges. Currently, there is a lack of safe and effective commercially accessible CDV vaccines suitable for non-domestic species. Occurrences of vaccine-induced distemper have arisen in domestic ferrets (*Mustela putorius furo*), black-footed ferrets (*Mustela nigripes*), European minks (*Mustela lutreola*), and various other wild carnivores. In the case of domestic ferrets, the key is the interference between attenuated live vaccines and maternal antibodies, causing inadequate defence in offspring born from vaccinated females [22]. Attenuated live vaccines also pose a risk of virulence reversion, leading to fatal infections in several wildlife species [23]. Safer choices such as inactivated virus, subunit or recombinant vaccines are recommended. DNA vaccines are being researched as an alternate approach to combatting CD, tackling the drawbacks of live attenuated vaccines [24]. Unfortunately, there are currently no safe vaccines for use in wildlife species [23].

CDV causes canine distemper (CD), a globally significant disease affecting both domestic dogs and wildlife species mainly in the order Carnivora. CDV also affects non-carnivore species such as collared peccaries (*Pecari tajacu*) [25], Japanese macaques (*Macaca fuscata*) [26], rhesus monkeys (*Macaca mulatta*) [27], wild boars (*Sus scrofa*), and Sika deer (*Cervus nippon*) [28]. The cross-species transmission potential of CDV is evident from sporadic spillovers into wildlife, leading to devastating mortality events and contributing to declines or near-extinction events in several wild animal populations including black-footed ferrets (*Mustela nigripes*) [29] and tigers (*Panthera tigris*) [30]. CD is an endemic disease, and numerous outbreaks have been documented across various regions globally [31]. In 2023, CD outbreaks were found in Galapagos Islands threatening the endangered Galapagos Sea Lion [32], in captive tigers in Thailand [33], and in Palm civets in India [34]. Recently, CDV was also detected in wild carnivores in multiple European countries, such as Italy, Hungary, Spain, Croatia, and Germany [35,36,37,38,39,40]. In Spain, CDV has been detected in Asturias, Lugo, Southwestern Andalusia, Serranía de Cuenca, Cantabric coast, Castilla y León, Aragón, and La Mancha [38,41,42,43,44,45]. Previous serological and viral nucleic acid studies show that different carnivores have been exposed to the virus cases, including red fox (*Vulpes vulpes*), American mink (*Neogale vison*), European polecat (*Mustela putorius*), stone marten (*Martes foina*), genet (*Genetta genetta*), Iberian lynx (*Lynx pardinus*), wolf (*Canis lupus*), European badger (*Meles Meles*), European mink (*Mustela lutreola*), pine marten (*Martes martes*), and European wildcat (*Felis silvestris*) [38,41,42,43,44,45,46]. Lineage studies in Spain are rare [38], resulting in a limited amount of research on the origin of CDV endemics. In recent years, the Wildlife Rehabilitation Centre (WRC) of Torreferrussa has found several positive cases in red foxes and European badgers. This has raised concerns about species conservation, as the Catalonia region is home to endangered species such as the Eurasian otter and the European polecat. The population of the European polecat shows a decreasing trend throughout Spain, especially near the Mediterranean, where the polecat population is quite scarce [46]. In Catalonia, the number of polecats, estimated by captures and sightings, was notably diminished after the invasion of the American mink [47]. In the areas where its presence has been confirmed, it has been estimated that the numbers could range between 100 and 200 individuals [48]. To protect the European polecat, a conservation project was put into place in Catalonia [48]. More breeding polecats will be released into the wild to enhance the wild population [49], which makes the investigation of CDV presence in Catalonia even more crucial.

Without an effective vaccine, managing CDV infection in European wildlife presents a challenging task. It calls for collaborative cross-border efforts, encompassing efficient monitoring, swift data collection, and the timely sharing of information [50]. Therefore, gathering epidemiological information such as health status, spreading patterns, and spatial distribution is a pivotal initial step in the control of CDV in wildlife. To control the spread of CDV in Catalonia or even globally, and unlock potential research value, this study may contribute to a clearer understanding of the virus’s circulation within multiple hosts and help evaluate the potential for interspecies transmission in a region of wildlife conservation. The current study aimed to achieve the following:

(a) Evaluate CDV presence and health status by identifying trends and potential novel infections in wildlife carnivores, including previously unreported species like the Eurasian otter;
(b) Conduct H gene sequencing on positive samples to compare strains with available lineages from NCBI, gaining crucial phylogenetic insights into CDV impacts on various species and predicting outbreak origins;
(c) Investigate the CDV spatial distribution among local wildlife populations by creating a geographical distribution map, contributing to a comprehensive understanding of transmission dynamics and facilitating targeted control strategies.

## 2. Materials and Methods

### 2.1. Study Area and Animal Tissues

Catalonia (32,114 km^2^), a region located in north-eastern Spain, encompasses four provinces characterised by diverse landscapes and has three distinct bioregions [51]: the Pyrenees Mountains, characterised by forests and alpine meadows, the bustling urban centres of the coastal regions, and the rolling plains of the interior. Among the principal rivers in Catalonia are the Ebro and their tributaries, the Ter in the North, and the Llobregat in the Centre, all of which run into the Mediterranean [51]. The four provinces within Catalonia are Barcelona, Girona, Lleida, and Tarragona. Adjacent to the north-eastern Spanish border, Catalonia is an autonomous community with its own governance and policies, including wildlife management.

Wild animals found sick or dead are collected by wildlife rehabilitation centres throughout Catalonia to investigate the causes of their illnesses and deaths. The animals analysed in this study (living and dead) were sent to the Wildlife Rehabilitation Centre of Torreferrusa (Barcelona). The American minks in this study were culled by wildlife rangers of Catalonia as part of invasive species management. Necropsies were performed on incoming carcasses and after ill animals had passed or were euthanised. Different animal tissues including the brain, lungs, liver, and kidneys, were taken and kept at −20 °C. In this study, 24 Eurasian otters (*Lutra lutra*), 28 red foxes (*Vulpes vulpes*), 15 European badgers (*Meles meles*), and 22 American minks (*Neogale vison*) collected from 2019 to 2023 were utilised. Carcasses were kept at −20 °C in the rescue centre, and after the selection of the most appropriate tissues, they were kept at −80 °C in a laboratory.

A database, including basic information such as species, time of collection, and organs collected, is available (Supplementary Information). Pictures of the American minks were taken during necropsies. For all other species, descriptions on intake information were recorded, including reasons for admission to the centre, causes of death, etc. In 10 cases with clinical signs or lesions suggestive of distemper, samples were sent to private veterinary laboratories to confirm diagnosis using PCR or histopathology techniques.

### 2.2. Nucleic Acid Detection and Analysis

For 23 animals (16 American minks, 6 red foxes, and 1 European badger), the kidney was chosen as the primary organ for CDV testing. Additionally, the lung was tested for the badger and four out of the six red foxes, with one of the red foxes also undergoing brain testing. For red foxes, European badgers, and Eurasian otters, the preferred organ for testing was the lung, followed by the brain. If the preferred organ was missing or compromised, the liver was used. In some suspicious cases where the kidney was analysed, the lung was also selected for secondary verification (Supplementary Information).

The selected animal tissues were homogenised using 1 mL of phosphate-buffered saline (PBS) solution. After thoroughly rinsing the tissue sample with PBS solution, the liquid was centrifuged at a centrifugal force of 13,000× *g* for 5 min. The supernatant was then carefully transferred without causing tissue fragmentation. The final suspension was kept at −80 °C.

Viral RNA extraction from the suspension was performed using TRIzol^®^ (Invitrogen, Fisher Scientific, S.L., Madrid, Spain) in accordance with the commercial protocol.

The specific forward (5′-CTTGCTTGCTATCACTGGAG-3′) and reverse (5′-TTTT GAAATCAAAGACATGG-3′) primers, designed for amplifying the H gene [52], were used to detect positive samples and characterise CDV lineage by sequencing. An amplicon of 484 bp RT-PCR was obtained using QIAGEN^®^ Onestep RT-PCR Master Mix with a 25 μL total reaction volume, which contained 1 μL of viral RNA, 1 μL each of the 10 μM primers, 1 μL of dNTP Mix (100 mM of each dNTP), 1 μL of One Step RT-PCR Enzyme Mix, 5 μL of 5× buffer, and RNase-free water. Initiating thermal cycling, a 30 min reverse transcription step at 50 °C was followed by initial 15 min PCR activation at 95 °C. The subsequent amplification phase consisted of 35 cycles: denaturation at 95 °C for 1 min, annealing at 52 °C for 30 s, and extension at 72 °C for 1 min. A final extension step was performed at 72 °C for 10 min. After RT-PCR, the amplicons were analysed via electrophoresis in 1.5% agarose gel solution with 0.5× Tris/Boric acid/EDTA (TBE) buffer. The gel ran at a voltage of 100 V/cm for 30 min and was subsequently stained with ethidium bromide for 30 min, and finally, the DNA bands were visualised using a UV lamp.

Positive and negative (RNase-/DNase-free water) controls were included during RNA extraction and in each RT-PCR test. The standard positive control was generated using a commercial vaccine (Nobivac^®^ DHP, MSD Animal Health, Salamanca, Spain). This vaccine contains live-attenuated CDV of the Onderstepoort strain at a concentration of 4.0 log_10_ TCID_50_. After resuspending the vaccine with 1 mL of RNase-/DNase-free water, a series of dilutions were prepared ranging from 10^0^ to 10^−4^.

The cDNA obtained was sent to the UAB sequencing service (Servei de Genòmica, UAB, Cerdanyola del Vallès, Spain) for Sanger sequencing. Sequences were assembled with Seqman Pro software (version 11.1.0.59) [53], aligned with Bioedit (version 7.2.5.0) [54], and analysed with MEGA11 (version 11.0.13) [55]. The distribution map was drawn with ArcGIS Online (https://www.arcgis.com/index.html, accessed on 21 August 2023) [56].

## 3. Results

In total, 18 out of 89 animals tested positive for CDV via RT-PCR (Table 1). Additional information about the positive cases can be seen in Table 2. Nine animals (eight red foxes and one European badger) showing neurological or respiratory clinical signs and five animals (four red foxes and one Eurasian otter) that passed away due to natural causes tested CDV-negative (Table S1).

These results showcase varying levels of CDV infections across the tested animal species. Red foxes had the highest positive percentage, followed by the European badger. Conversely, American minks exhibited a lower rate, while Eurasian otters did not show any positive cases.

Two red foxes tested positive for CDV in their kidney tissue but tested negative in their lung tissue. An unrelated red fox tested positive in its brain and lung tissue but tested negative in its kidney tissue.

**Figure 1:**
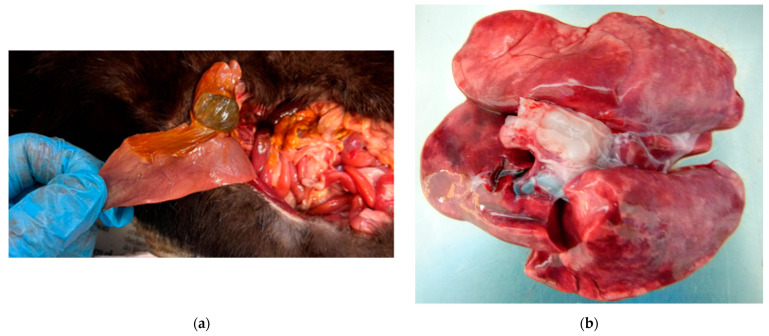
Available pathological pictures of two positive cases: (**a**) liver of a positive American mink (*Neogale vison*), No. 116, showing yellowish discolouration; (**b**) lungs of the European badger (*Meles meles*), No. 151, showing macroscopic signs of pneumonia.

Figure 1 illustrates images corresponding to two positive cases for which photos were taken during necropsy. Figure 1a shows a yellowish discoloured liver from No. 116. Figure 1b shows the lungs of the positive case, No. 151. In one case (No. 166), histopathology was performed at a private veterinary laboratory, which confirmed the suspicion of distemper. Unfortunately, we did not have access to microscopic images or their descriptions.

**Figure 2:**
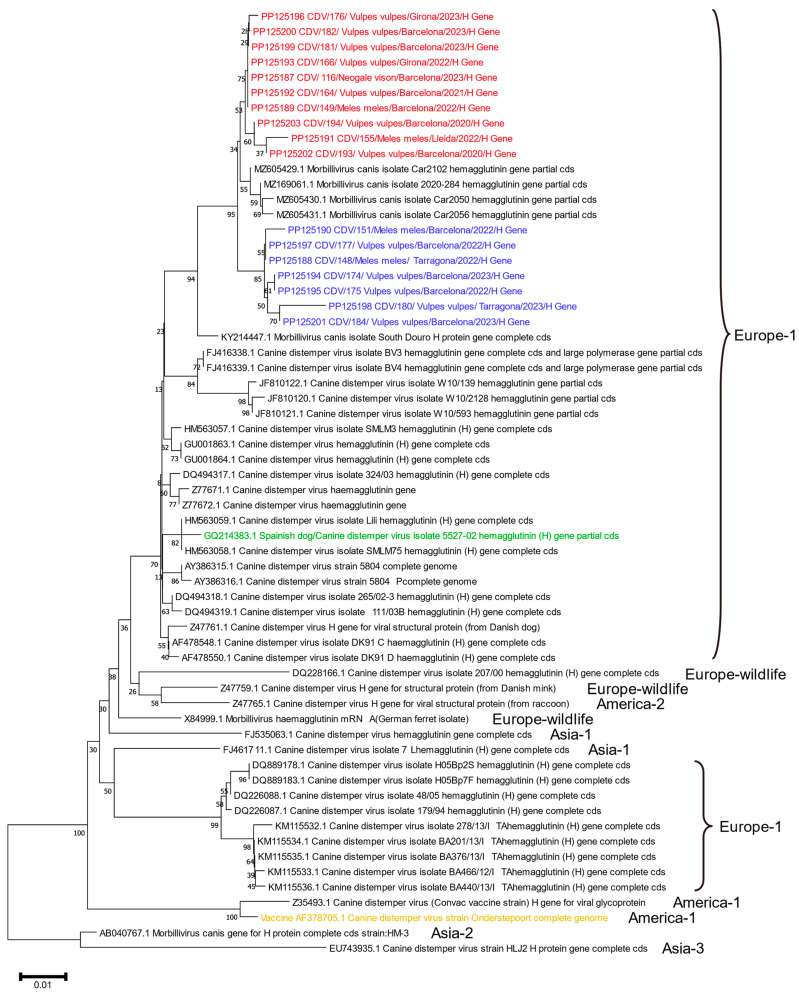
Phylogenetic tree of CDV isolated in the study with other related CDV virus information [1,2,44,52,58,59,60,61,62,63,64,65,66] with lineage types marked. The tree was built up using the neighbour-joining method and the Tamura–Nei substitution rate. The confidence of the internal branches was inferred with 1000 bootstrap pseudo-replicates. The strains from this study are named NCBI_reference CDV/ID/Species/ Province /Year/H gene. They are coloured in red and blue according to different genetic similarities. The strain (GQ214383.1) isolated from a Spanish dog entering Austria is coloured green and the Onderstepoort strain is coloured yellow. Lineage information of all the strains is written on the right.

The phylogenetic tree (Figure 2) shows the isolates detected in this study and their relationship with CDV lineages. All of the isolates reside within the Europe lineage. RNA from a virus (GQ214383.1) isolated from a Spanish dog entering Austria [57] and the Onderstepoort strain contained in the vaccine used as positive control were also included in the phylogenetic tree to gain more information, as were all wildlife CDV sequences available in NCBI database [1,2,44,52,58,59,60,61,62,63,64,65,66]. There were no other sequences from dogs in Spain available in the NCBI database. On the other hand, the viral strain derived from the vaccine Onderstepoort belongs to the American-1 lineage, representing a completely distinct genetic lineage. Also, in contrast to the strains from this study, the mutation number in Onderstepoort strains is 23 and the mutation percentage is 4.76%.

**Figure 3:**
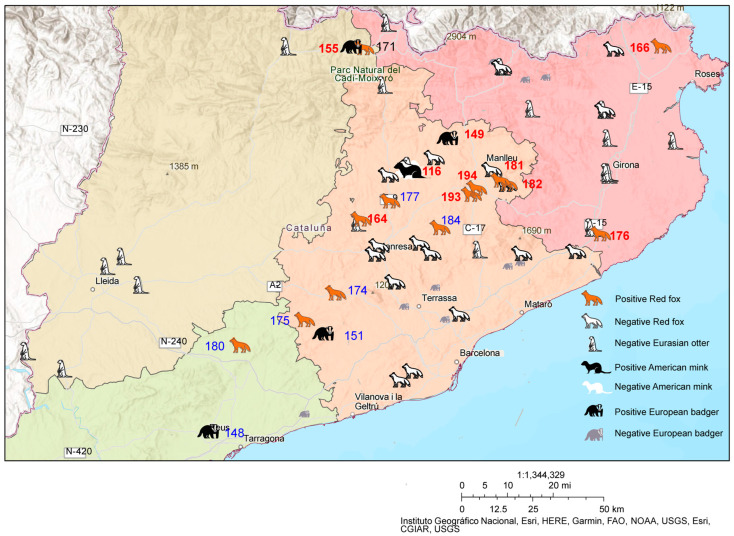
Distribution of carnivore samples in Catalonia. Detailed information can be seen in Table 2. Positive cases were marked with their ID number coloured to distinguish different branches in Figure 2 (red and blue). The strain failed to be sequenced (171) is coloured black.

Figure 3 illustrates the geographical distribution of the 89 animals sampled in Catalonia, with a clear differentiation between species and their CDV-positive/-negative status. Additionally, based on the two branches identified in Figure 2, distinct colours were employed to depict these branches on the map.

The majority of the collected animal samples were from the Barcelona province (51), followed by Girona (24), Lleida (10), and Tarragona (4).

Genetic divergence between two branches from Figure 2 is visualised in Figure 3; one branch (red colour) is prominently distributed in the northern region, while the other (blue colour) is distributed in the southern region.

## 4. Discussion

Canine distemper is a severe disease in domestic dogs that also affects a wide range of wild animals, especially carnivores (canids and mustelids). The infection has been detected worldwide and has the potential to cause serious problems for endangered species.

In recent years, several mortality cases in red foxes caused by CDV have been observed in Catalonia, but information has been lacking on the lineage, possible origin of the isolates, and distribution of the infection in wildlife. Samples from a variety of wild carnivore species were used to further understand the dynamics of CDV in Catalonia. In this study, diagnosis by means of specific molecular techniques for CDV RNA was applied to detect the circulation of the virus in dead or sick animals arriving at the WRC of Torreferrussa located in Catalonia. This introduces a potential sampling bias, as this study is not the result of random sampling, which in turn will affect the objectivity in reflecting the actual prevalence of CDV. The findings provide a percentage of positive CDV cases but cannot be considered a direct reflection of CDV’s true prevalence in wildlife due to the non-random sampling nature. Another fact to consider is that the American minks collected underwent culling as part of the invasive species control campaign in this region, were apparently healthy, and were killed once captured.

Among the CDV-positive animals, 12 of them arrived at the WRC with clinical signs compatible with CDV infection, and 4 animals had trauma identified as the ultimate cause of death. These casualties of trauma could be presumptively related with the neurological disease associated with CDV infection [67]. Besides the description demonstrated in the Table 2, more information can be seen in Table S1. In total, 45 animals exhibited different kinds of sickness, while 41 animals were dead from trauma or gunshot, or were euthanised, and 3 animals were unknown. For 15 animals exhibiting potential CDV infection signs, 3 were tested negative in the lung for CDV: 1 red fox (No. 165) with neurological signs, another red fox (No. 170) with pneumonia, and a badger cub with a pale liver (the liver sample was lost). This implies that in these hosts, canine distemper tends to show more obvious clinical signs, and most animals displaying pneumonia and a neurological picture are diagnosed with canine distemper.

In total, 89 carnivores (foxes, badgers, American minks, and European otters) were included in this study, and 18 were positive in one or more tissues (Table 1). In some cases, different tissues showed different results within the same animal. This diversity can be attributed to the systemic nature of CDV infection, whereas the virus can target and replicate in various organs throughout the body. As the disease progresses, CDV may accumulate differently in various organs. This intriguing pattern suggests potential variations in the tissue tropism of the virus within individual animals, affecting the choice of tissues tested. In previous studies, all studied species exhibited primary susceptibility in their CNS, lungs, and lymphoid tissues [68]. It was also found that the highest levels of viral antigens were detected in different parts of the CNS among various species [38]. In general, this phenomenon emphasises the need for a comprehensive approach in sampling multiple tissues to accurately assess the extent and impact of CDV infection in an affected host. However, in this study, time and resources were limited, and testing all the available tissues in every animal was not feasible. Initially, not every organ was collected for every animal in the beginning necropsy stages. The brain was not the primary organ choice either, as prolonged storage at −20 °C makes RNA virus preservation challenging in decomposing brain tissue. Similar issues were also found in a previous study, as fresh and better quality tissues might exhibit better results [38]. In this study, animal carcasses were kept at −20 °C, which is not the best preservation temperature for viral RNA. Accordingly, the lungs became the primary choice for tissue analysis due to their relatively well-preserved structure. For American minks, kidneys and lungs were chosen since the pictures were readily available from the necropsy sampling stage. In other words, the possibility of a few false negatives cannot be ruled out, as more positive cases may have been identified if the tissues tested would have been properly preserved.

The high percentage of positive cases (44.8%) in red foxes raises concerns. The red fox (*Vulpes vulpes*) can be the biggest reservoir in wildlife of CDV in the environment. Recent phylogenetic analysis suggests that wild animal species such as foxes in Denmark could potentially contribute to the transmission of CDV to farmed minks. The virus could then persist within the host’s wild animal population during outbreak periods [69]. The results show a potential impact of CDV on the health and survival of red foxes, as well as its potential implications for the broader ecosystem. The European badger and red fox can cohabit during reproductive seasons [70]. This interaction between the European badger and the red fox introduces a new purpose to observe the spread of CDV. This could be a contributing factor in explaining why the European badger also demonstrates a relatively high percentage (28.6%) of infected animals, following the red fox. The observed higher detection rate in red foxes and the moderate rate in European badgers highlight them both as potential reservoir species for CDV.

The absence of CDV detection in Eurasian otters in this study may suggest the possible low susceptibility or limited exposure for this species. An investigation focused on detecting CDV presence in the Eurasian otter population in Hungary from 2000 to 2021 found that out of 339 samples, merely 2 tested positive for CDV [37]. This limited prevalence could imply that CDV is not extensively prevalent within the Eurasian otter population.

A serological survey on CDV in mustelids collected from March 1996 to March 2003 in South-Western France showed that the antibody prevalence in the free-ranging American minks was 5% in 112 animals [46]. Another serological survey on CDV in 87 wild American minks in Argentina revealed a seroprevalence of 2.3% [71]. Nevertheless, the actual CDV prevalence is lower than the antibody prevalence. In this study, the positive percentage of CDV was 4.5% in the 22 American minks tested. Although this sample size is not large enough to reflect the CDV infection status of American minks in Catalonia, these individuals were not found sick or dead, so this might suggest that the American mink could be a potential reservoir species in the environment, the same environment that is shared with the Eurasian otter. Although the Eurasian otter in this study did not show any positive results, continuous surveillance studies should be carried out in this near-threatened (NT) species.

Concerning animal conservation, apart from the animals included in this study, the European polecat (*Mustela putorius*) stands out as one of the most endangered species within the *Mustelidae* family in Europe. Native to Spain, this species encounters a conservation challenge as its population exhibits a declining trend across the country [72]. The European polecat occasionally lives in the abandoned burrows of red foxes and European badgers [73]. This also provides a possibility for CDV to be transmitted via the sharing of caves and other living spaces. In addition, there is an overlap between the polecat and American mink’s spatial and trophic niches [47], which might also contribute to the transmission of CDV. Recalling the positive percentage results from this study, more attention needs to be paid to this species.

Figure 1 shows a liver lesion in a positive case of an American mink that was tested positive in the liver tissue, but it cannot be confirmed that the lesion was caused by CDV. The animal could have been carrying other pathogens that may have caused this liver condition. In a pathological study on wild felids, liver lesions were studied on a histological level [74]. Liver lesions like the swelling of hepatocytes with individualised, severe sinusoidal congestion and vacuolisation of the hepatic cytoplasm were found [74]. This kind of liver lesion is not different from that in other species reported [75,76]. To prove that the liver lesion in our study was caused by CDV, histological studies are needed, which unfortunately were not performed due to time and resource restrictions.

As shown in Figure 2, all of the isolates in this study belong to the European-1 lineage. The results showed small genetic differences between the viral lineages isolated from wildlife species (marten, red fox, and badger) in the Asturias region (the North of Spain) noted from a previous study [38]. Additionally, our isolates were categorised into two branches with the strains from the study of Oleaga et al. [38] situated in between, which are closer to the red branches. The isolate outside of Spain that is most closely related to the CDV isolates in this study comes from one strain in a wolf from Portugal (KY214447.1) [77]. This was also confirmed via the blasting of the strains in this study in NCBI. This suggests that the genetic lineage in the Catalonia region remains localised, and there is no significant divergence in wild carnivores within the entire Iberian Peninsula yet. In other words, the viruses in this study are unlikely to have been introduced suddenly from outbreaks caused by foreign strains.

The significant genetic difference between a Spanish canine isolate (GQ214383.1) and the CDV isolates in this study make it challenging to infer a direct connection in the transmission chain of CDV between dogs and free-ranging wild carnivores. The strains affecting dogs are most likely not the same as the ones in the wildlife population. In a CDV spillover study in Africa, no evidence was found that dogs introduced CDV into wild carnivores [78] even though dog populations in Africa are speculated to be potential CDV reservoirs [79,80]. In Catalonia, the chance that hunting or domestic dogs interact directly or indirectly with wild carnivores cannot be ruled out. However, we have not found any Spanish isolate in the NCBI database, and the only strain included in the phylogenetic tree was described 12 years ago [57]. Furthermore, the CDV strains isolated in Catalonia are very much unlikely related to a release from an attenuated vaccine into the environment.

In Figure 3, possible CDV transmission among different species can be observed. Three out of four European badger cases showed a geographical correlation with red fox cases, supporting the theory that European badgers might be more susceptible to CDV infection due to their sharing of dens with red foxes [70]. This fact also highlights the need to pay attention to the CDV situation in European polecats, a protected wild species, as they also share dens with these two species.

Studying genetic similarity (Figure 2), it is observed that different individual animals and species share the same viral strain. Two red foxes (No. 174 and No. 175) share a common strain, while an American mink (No. 116), a red fox (No. 181), and a European badger (No. 149) share another common strain. This shows strong evidence for CDV rapid transmission across distances and between different species, indicating outbreaks in these two regions of Catalonia. Another notable observation is that the viral sequences from a red fox in central Barcelona (No. 177) and a European badger in Tarragona (No. 148) are the same, although the hosts are different species and geographically distant from one another. This raises the possibility of a rapid and widespread transmission of the virus happening in southern Catalonia, limiting the time for mutations of CDV. To support this long-distance spread, CDV could be sustained via the interplay of various subgroups, each undergoing sporadic yet asynchronous outbreaks of CDV [78]. Moreover, the rapid and robust immunising characteristics of CDV infection may imply the necessity of substantial populations of susceptible hosts for the persistence, circulation, and transmission of the virus [81,82]. CDV spreads swiftly among juveniles, depleting susceptible individuals. Diminished herd immunity over time, coupled with new susceptible juveniles, may lead to fresh outbreaks [78]. This suggests that species that have big populations and high contact with other groups and species, such as the red fox and the European badger, play an important role in spreading CDV.

## 5. Conclusions

The health situation of wild carnivores is not optimistic, with a high risk of CDV infection and transmission. The percentage of positive cases is relatively high in red foxes and European badgers, leading to high CDV exposure in an environment where threatened and endangered species live. On the distribution map, there are distant animal individuals and different species sharing the same strain, along with rather distant animals sharing very similar strains, which might point to outbreaks of CDV in these areas.

In conclusion, this study shows the complex dynamics of CDV infections within the wildlife populations in Catalonia. The findings emphasised the urgency of addressing the potential impacts of CDV on both the health of local species and the ecosystem. Moreover, endangered species such as the European polecat should be included in future studies.

## Figures and Tables

**Table 1:** CDV positivity percentage in different carnivores and positive ratios in different organs.

Species	Percentage (Positive/Total Animals)	Number of Tissues Tested Positive/Total Tissues Tested				
		Lung	Liver	Brain	Kidney	Total
Red fox (*Vulpes vulpes*)	44.8% (13/29)	9/26	0/1	1/1	4/6	14/34
European badger (*Meles meles*)	28.6% (4/14)	4/14	0	0	1/1	5/15
American mink (*Neogale vison*)	4.5% (1/22)	0/0	1/6	0	0/16	1/22
Eurasian otter (*Lutra lutra*)	0% (0/24)	0/23	0	0/1	0	0/24
Total	20.2% (18/89)	13/63	1/7	1/2	5/23	20/95

**Table 2:** Information of CDV-positive animals, including the province where they were captured, the date they received necropsy, the NCBI reference for sequence information, and the description of the animals when they arrived at the WRC. NA: ID 171 failed to sequence and is not available.

ID	Species	Province	Year	NCBI Reference	Description of the Case
116	*Neogale vison*	Barcelona	2023	PP125187	Euthanised (culled)
148	*Meles meles*	Tarragona	2022	PP125188	Trauma (Hit by car) ^1^
149	*Meles meles*	Barcelona	2022	PP125189	Distemper (neurological signs and pneumonia) ^2^
151	*Meles meles*	Barcelona	2022	PP125190	Distemper (neurological signs and pneumonia) ^2^
155	*Meles meles*	Lleida	2022	PP125191	Trauma (Hit by car) ^1^
164	*Vulpes vulpes*	Barcelona	2021	PP125192	Distemper (pale organs) ^1,2^
166	*Vulpes vulpes*	Girona	2022	PP125193	Distemper (pneumonia and hepatitis) ^1,3^
171	*Vulpes vulpes*	Lleida	2022	NA	Distemper (pneumonia) ^1,2^
174	*Vulpes vulpes*	Barcelona	2023	PP125194	Distemper (neurological signs and pneumonia) ^2^
175	*Vulpes vulpes*	Barcelona	2022	PP125195	Undetermined ^1^
176	*Vulpes vulpes*	Girona	2023	PP125196	Distemper (neurological signs and pneumonia) ^2^
177	*Vulpes vulpes*	Barcelona	2021	PP125197	Distemper (neurological signs and pneumonia) ^2^
180	*Vulpes vulpes*	Tarragona	2023	PP125198	Distemper (pneumonia) ^1,2^
181	*Vulpes vulpes*	Barcelona	2023	PP125199	Trauma (Hit by car) ^1^
182	*Vulpes vulpes*	Barcelona	2023	PP125200	Trauma (Hit by car) ^1^
184	*Vulpes vulpes*	Barcelona	2023	PP125201	Distemper (conjunctivitis and pneumonia) ^2^
193	*Vulpes vulpes*	Barcelona	2020	PP125202	Distemper (neurological signs and pneumonia)
194	*Vulpes vulpes*	Barcelona	2020	PP125203	Toxoplasmosis (neurological signs)

^1^ Dead animal upon admission. ^2^ Confirmed diagnosis of distemper via PCR. ^3^ Confirmed diagnosis of distemper via histopathology.

## Data Availability

All data generated or analysed during this study are included in this published article (and its Supplementary Information files).

## Supplementary Materials

The following supporting information can be downloaded at https://www.mdpi.com/article/10.3390/ani14030436/s1: Table S1: Information of all the animals from this study, including species, ID, the place where they were found, their gender, the date they were necropsied and tested, the description of the animals when they arrived at the WRC, and test results and details.
